# Left Pulmonary Artery Aneurysm Secondary to Metastatic Lung Sarcoma: A Case Report

**DOI:** 10.7759/cureus.44321

**Published:** 2023-08-29

**Authors:** Muhammad Abdullah Javed, Kaleem S Ahmed, Mohammad Bin Pervez, Saira Fatima, Saulat Hasnain Fatimi

**Affiliations:** 1 Medical College, Aga Khan University, Karachi, PAK; 2 McCormick School of Engineering, Northwestern University, Evanston, USA; 3 Surgery, University of Toronto, Toronto, CAN; 4 Cardiothoracic Surgery, Aga Khan University, Karachi, PAK; 5 Histopathology, Aga Khan University, Karachi, PAK

**Keywords:** pulmonary artery pseudoaneurysm, aneurysm, lung sarcoma, pulmonary artery aneurysm, metastatic lung cancer

## Abstract

Aneurysms are characterized by focal dilation of the blood vessel wall due to weakening. The involvement of two layers of the vessel wall is classified as a pseudoaneurysm while the involvement of all three layers is called a true aneurysm. Involvement of neoplastic lesions is rare, but the few reported cases have been associated with pulmonary artery pseudoaneurysms as opposed to true pulmonary artery aneurysms (PAAs). Our case of a true left PAA of a patient with metastatic sarcoma of the lung shows an association that has previously not been reported to the best of our knowledge.

## Introduction

Aneurysms are clinical disorders characterized by focal dilation of the blood vessel wall due to weakening [1,2]. The involvement of two layers of the vessel wall is classified as a pseudoaneurysm while the involvement of all three layers is called a true aneurysm [3]. The occurrence of pulmonary artery aneurysms (PAAs) or pulmonary artery pseudoaneurysms (PAPAs) is etiologically classified as congenital or acquired with the latter being more common. Involvement of neoplastic lesions is rare, but the few reported cases have been associated with PAPA as opposed to PAA [1]. They are due to primary lung cancers or metastatic cancers to the pulmonary artery and act via vessel wall erosion and direct tumor invasion. In this report, we present a case of a true left PAA of a patient with metastatic sarcoma of the lung and this association has not been previously reported to the best of our knowledge.

This article was previously presented as an abstract at the Chest Virtual Congress on June 26, 2020, and at Aga Khan University's 12th Health Sciences Research Assembly from December 14 to 18, 2020.

## Case presentation

A 53-year-old gentleman presented to a tertiary care hospital in August 2017 with a dry cough and low-grade fever. He had previously been in his usual state of health until one week prior when he developed these symptoms along with chest pain. He also had a weight loss of 4 kg during this time. He reported visiting multiple local physicians since the onset of his symptoms and a CT scan performed upon the request of his primary care physician incidentally was suggestive of two PAAs (7.7 x 4.0 cm and 7.4 x 8.0 cm) surrounding thrombosed sections of the left pulmonary artery (PA). A review of systems revealed that he had a decreased appetite but normal sleep and bowel habits. His past medical history was significant for diabetes mellitus (DM) and hypertension (HTN). He was not taking any medications at the time of admission.

On examination, he was tachycardic, but his other vitals were normal. A general physical examination revealed no significant findings. Cardiovascular, abdominal, and neurological examinations were normal while the respiratory exam revealed decreased air entry on the left side. Laboratory findings at the time of admission were suggestive of iron-deficiency anemia and revealed a significantly elevated erythrocyte sedimentation rate (ESR) of 63 mm/hr. Glycosylated hemoglobin (HbA1c) was elevated at 8.90% and fasting glucose was also deranged. Blood urea nitrogen (BUN) was mildly elevated at 30.7 mg/dl while creatinine was within normal ranges at 1.05 mg/dl (Table 1). A provisional diagnosis of a left lung mass and left PAAs was made.

**Table 1:** Laboratory parameters

Laboratory parameters	Results	Reference values
Erythrocyte sedimentation rate (ESR)	63 mm/hr	0-20 mm/hr
Hemoglobin A1c/glycated hemoglobin (HbA1c)	8.90%	Diabetes ≥ 6.5%
Blood urea nitrogen (BUN)	30.7 mg/dl	6-20 mg/dl
Creatinine	1.05 mg/dl	0.9-1.3 mg/dl

For preoperative evaluation, the patient underwent pulmonary function testing (PFT). However, the values are unfortunately not present in our electronic health record system since they were likely done from an outside laboratory. Due to resource limitations and financial constraints, a bronchoscopy was performed preoperatively on the table to save costs. An elective left pneumonectomy and subsequent tissue biopsy were performed under general anesthesia. Intraoperative findings revealed extensive inflammation and venous congestion of the left lung. A tissue sample consisting of two fragments (aggregate size of 1.5 x 1.0 cm) sent for a frozen section revealed a neoplastic lesion. Immunohistochemical stains showed WT1 cytoplasmic positivity. Large biopsy samples were taken and remnant tissue from the pericardial surface was also removed. There were no intraoperative complications. A gross examination of the biopsy samples revealed a tumor 7 x 7 x 6 cm in size. Histopathology of the left lung section revealed a neoplastic lesion arranged in sheets. An immunohistochemical stain was also performed (Table 2). Pleural surface involvement was also found. Terminal bronchioles were intact and the bronchial resection margin was tumor-free, which was grossly 2.8 cm away. Hilar, mediastinal, and level VII lymph nodes were also tumor-free. The inferior pulmonary vein margin revealed a tumor fragment and no venous wall was identified in the specimen. The pericardial membrane margin revealed fibrocollagenous tissue invaded by the tumor. The final histopathological diagnosis confirmed a poorly differentiated malignant neoplasm most likely to be a high-grade sarcoma or sarcomatoid carcinoma.

**Table 2:** Histopathological markers on frozen section and biopsy

Histopathological marker	Reactivity
ASMA (α-smooth muscle actin) patchy	Positive
WT1 (Wilms tumor suppressor gene 1) cytoplasmic	Positive
CD31 (cluster of differentiation 31)	Negative
CD34 (cluster of differentiation 34)	Negative
ERG (erythroblast transformation specific/ETS-related gene)	Negative
Epithelial membrane antigen (EMA)	Negative
Cytokeratin 5/6	Negative
Cytokeratin AE1/AE3	Negative
Cytokeratin CAM 5.2 (cell adhesion molecule 5.2)	Negative
CD30 (Ki-1) (cluster of differentiation 30)	Negative
Pan B (CD20) (cluster of differentiation 20)	Negative
Pan T (CD3) (cluster of differentiation 3)	Negative
TTF1/p63 (thyroid transcription factor 1/transformation related protein 63)	Negative
34-beta-E12 (cytokeratin 34-beta-E12)	Negative
Ber-EP4 (epithelial cell adhesion molecule/EPCAM)	Negative
Calretinin	Negative
Desmin	Negative
HMB45 (human melanoma black 45)	Negative
Myogenin	Negative

Postoperatively, he was started on intravenous (IV) antibiotics and IV fluids. Pain management and chest physiotherapy were done as well. Oncology was taken on board and he was started on adriamycin and ifosfamide. His pre-discharge labs were normal, and he was discharged on postoperative day five. In the postoperative period, he had one readmission for pleural effusion and empyema at the six-month mark, for which he electively underwent video-assisted thoracic surgery (VATS) and decortication. He remained stable postoperatively and was discharged on postoperative day three. He was then started on adriamycin/ifosfamide cycle therapy as an oncology out-patient.

In the one-year follow-up after his VATS, he had one readmission for sepsis, developed brain metastases in April 2018, and was started on radiotherapy. On his last follow-up in January 2019, around 1.5 years after his initial presentation, he was doing fine with no complications; however, on a phone follow-up on a later date, it was found that the patient had expired. The date of the phone follow-up was not recorded, so it can be assumed that he expired sometime after January 2019.

## Discussion

PAAs are clinical abnormalities characterized by focal dilation of the blood vessel wall due to weakening [1,2]. Involvement of all three layers of the vessel wall is classified as a true aneurysm while involvement of any two layers is classified as a pseudoaneurysm [3]. As such, the latter is considered to have a higher risk of rupture, with reported mortality rates varying from 50-% to 100% [2].

Clinical manifestations range from asymptomatic [4] to shortness of breath, hemoptysis, chest pain, palpitations, and episodes of syncope [5]. Symptoms of extrinsic bronchial compression may occur in patients with large PAAs or PAPAs. The confirmatory diagnosis of PAPAs or PAAs is done radiologically due to the non-specificity of symptoms, and it is often an incidental finding on CT [2], which shows a greater predilection for the left pulmonary artery. Aneurysms typically appear as a lung nodule or hilar enlargement on radiographs and the diagnosis is confirmed using a CT scan with contrast. On CT, an aneurysm is diagnosed if dilation causes the diameter to exceed the upper limit of normal, which is 29 mm for the main pulmonary artery and 17 mm for the right interlobar artery [1]. Similarly, in our patient, the aneurysms measured 7.7 x 4.0 cm and 7.4 x 8.0 cm, which is above the normal diameter limit. Moreover, lung metastases typically present with multiple PAAs or PAPAs and this correlates with our findings, as CT revealed two PAAs in the left pulmonary artery in our patient [4].

PAAs or PAPAs are etiologically classified as congenital or acquired. Congenital causes are due to congenital heart defects or connective tissue abnormalities. The former’s suggested pathophysiology relates to increased hemodynamic sheer stress that is often associated with left-to-right shunts [2,6].

Acquired PAAs or PAPAs are sub-classified into infectious, vasculitis, pulmonary artery hypertension, chronic pulmonary embolism, chronic inflammatory lung disease, neoplasms, trauma, and iatrogenic causes [2]. Involvement of neoplastic lesions is rare, and most reported cases have been associated with PAPA [1] as opposed to PAA. They are due to primary lung cancers or metastatic cancers to the pulmonary artery and act via vessel wall erosion and direct tumor invasion, with the latter being less common. In the past, pseudoaneurysms have been diagnosed in patients with bronchial carcinoma [7], squamous cell carcinoma of the lung [8], and angiosarcoma [9], while PAAs have typically been reported in patients with ipsilobar non-small cell lung cancer [10]. Primary pulmonary artery tumor presenting as PAA is rare; however, it has been reported in patients with pulmonary artery sarcoma and pulmonary artery histiocytoma [11,12]. Our report varies from this pattern as it presents a novel case of a metastatic sarcoma causing a true left PAA as opposed to a PAPA and this association has not been previously reported to the best of our knowledge.

## Conclusions

Acquired PAAs and PAPAs can be attributed to multiple causes; however, association with malignancy is rare with a majority of the reported cases presenting with PAPAs. This is either due to primary lung cancers or metastatic cancers to the pulmonary artery causing wall erosion. The association reported above is unique, as it narrates the case of a 53-year-old gentleman who presented with a true PAA as opposed to PAPA secondary to metastatic sarcoma of the lung, an association, which, to the best of our knowledge, has not been reported yet.
